# Performance of landscape composition metrics for predicting water quality in headwater catchments

**DOI:** 10.1038/s41598-019-50895-6

**Published:** 2019-10-08

**Authors:** Linda R. Staponites, Vojtěch Barták, Michal Bílý, Ondřej P. Simon

**Affiliations:** 1 Faculty of Environmental Sciences, Czech University of Life Sciences Prague, Kamýcká 129, Praha, Suchdol 165 00 Czech Republic; 2 T. G. Masaryk Water Research Institute, Podbabska 30, 160 00 Prague 6, Czech Republic

**Keywords:** Hydrology, Limnology

## Abstract

Land use is a predominant threat to the ecological integrity of streams and rivers. Understanding land use-water quality interactions is essential for the development and prioritization of management strategies and, thus, the improvement of water quality. Weighting schemes for land use have recently been employed as methods to advance the predictive power of empirical models, however, their performance has seldom been explored for various water quality parameters. In this work, multiple landscape composition metrics were applied within headwater catchments of Central Europe to investigate how weighting land use with certain combinations of spatial and topographic variables, while implementing alternate distance measures and functions, can influence predictions of water quality. The predictive ability of metrics was evaluated for eleven water quality parameters using linear regression. Results indicate that stream proximity, measured with Euclidean distance, in combination with slope or log-transformed flow accumulation were dominant factors affecting the concentrations of pH, total phosphorus, nitrite and orthophosphate phosphorus, whereas the unweighted land use composition was the most effective predictor of calcium, electrical conductivity, nitrates and total suspended solids. Therefore, both metrics are recommended when examining land use-water quality relationships in small, submontane catchments and should be applied according to individual water quality parameter.

## Introduction

It has been widely acknowledged that the ecological integrity of streams and rivers is intrinsically linked to the surrounding landscape^1–3^. Riverine systems are amongst the most productive and biodiverse ecosystems^4^, yet extreme anthropogenic pressure has threatened the essential goods and services provided by tributaries^5^. The protection of freshwater resources and ecosystems requires an understanding of the impacts from the encompassing landscape. Although land use-water quality interactions have been extensively researched, a comprehension of such relationships remains a complex endeavor. To discern the effects of land use on water quality, initial investigations frequently employed land use composition (i.e., the proportion of each land use category) as a predictor of stream condition (e.g.^6,7^). While the composition of land use plays a crucial role on water quality, this rudimentary measure assumes that each proportion imposes an equal influence^8^. Recently, the importance of spatial scale and topography has been corroborated in the contemporary understanding of land use-water quality interactions^8,9^. Nevertheless, the intricate patterns and natural gradients of a terrestrial landscape, as well as scale-dependent mechanisms, make it difficult for empirical models to be assessed^2^. The integration of spatially-explicit landscape features and processes with land use data is crucial for providing more accurate information on how land use can impact concentrations of water quality parameters (WQP).

With the application of Geographic Information System (GIS) technologies, broadly-applicable weighting schemes have been established as methods to consider the spatial and topographic components of individual land use types on stream condition. Under the assumption that land located close to the stream generally has a larger influence on water quality than land located further away^8,10–14^, distance-weighted metrics have been implemented into studies to account for the spatial proximity of land use^10,11,15–18^. In this method, a distance decay function is used, assigning weights to observations based on the hydrologic distance to the stream or sampling point to elucidate the inordinate impact of land situated close to the source. Additionally, flow accumulation has been incorporated into distance-weighted metrics on the basis that areas and pathways of concentrated flow have a higher tendency to generate runoff^16,19^. The concentration of overland runoff within each land use category is weighted according to the flow accumulation value determined by flow direction and preferential flow pathways from upslope areas. Such metrics are particularly suitable for examining the combined effects of land use position and hydrological processes^19^. Although studies have concluded that spatially-explicit methods can improve predictions of stream conditions and are more effective than non-spatial methods^3,11,17–19^, the best weighting schemes were often determined according to the ecological response of various aquatic species assemblages. Little is known about optimal metrics for predicting the effects of land use on individual chemical parameters. Furthermore, it is unknown if the inclusion of additional variables and functions can enhance the accuracy of predictive models.

Located in Central Europe, the headwater catchments of South Bohemia, Czech Republic are a typical example of a submontane landscape, characterized by mainly forests and meadows. Headwater streams and catchments are particularly important for provisional ecosystem services (e.g., drinking water extraction) and the protection of biodiversity (e.g., nature reserves and core zones of national parks)^20^. Tributaries act as both receptors and conveyors of landscape fluxes^21^, allowing upstream land use activity to influence the entire river continuum^22,23^. The development of strategic management plans within headwater catchments is, thus, imperative for improving downstream conditions. Quantifying and comparing the predictive power of empirical models using various landscape composition metrics can provide a comprehensive evaluation of the impacts of land use on water quality and better aid in the identification of landscape processes affecting this relationship^19^.

In this work, various landscape composition metrics are applied and augmented to explore the predictive power of the catchment-scale landscape on the concentration of eleven WQP within headwater streams. The main objectives of this study are to (1) examine the variations in performance between landscape composition metrics, (2) investigate how the incorporation of stream proximity, slope and flow accumulation can influence the predictive ability of models, and (3) identify which landscape composition metric explains the most variation in water quality data.

## Methods

### Study area

The headwater streams of the Upper Vltava River Basin, located in the South-West of the Czech Republic, originate within the low-range Šumava Mountains which border Germany and Austria. Due to its oligotrophic waters, this region provides refuge for many rare aquatic species^24,25^ and harbors sources of drinking water^26^. The region consists of a temperate climate with a mean annual precipitation of approximately 1400 mm and a mean annual temperature of about 4 °C^27^. The majority of the study area is included in the European system of protected areas (Natura 2000), leaving the landscape in a relatively undisturbed, semi-natural state^28^.

**Figure 1:**
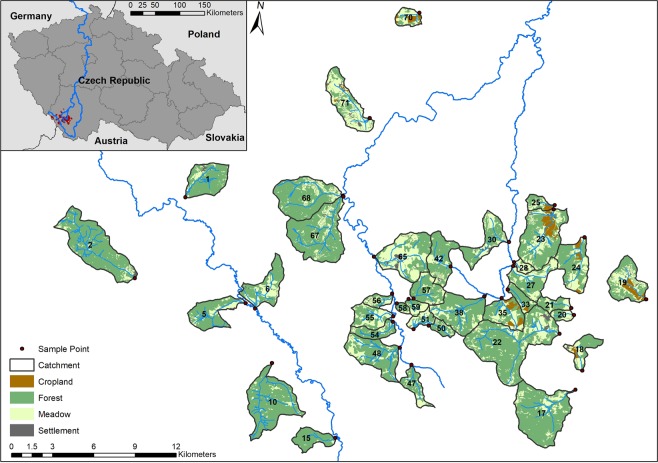
Selected catchments, sampling points and land use pattern with the main tributaries draining the catchments.

Thirty seven headwater catchments were selected, ranging in size from 0.61 km^2^ to 18.85 km^2^ with stream orders ≤3 (Strahler method) (Fig. 1). The topography within catchments varies from hilly mountain ranges to fairly flat areas with elevations ranging from ~530 m a.s.l. to 1288 m a.s.l. and sampling points averaging ~708 m a.s.l. (±104 SD), allowing for a representative survey of the study area. Forests are the predominant land use within most catchments, comprised mainly of spruce or a mixture of spruce, pine and broadleaf forest stands^27,29^, while meadows used for grazing and hay production are also prevalent. Intensive meadows can constitute as sources of eutrophication^30^, however, liquid fertilization of grasslands has been decreased or discontinued within parts of this region^31,32^. As with many other border regions within the Palearctic, this sparsely populated area has experienced a gradual recession in farming due to barren soils unsuitable for agricultural intensification^33^. Over time, extensive agriculture has been replaced by meadows, with only a small extent of crop fields remaining on the foremost fertile soils^34^. In order to focus on the primary land use types within the region, only catchments with at least 77% of forested and grassed composition, and without significant point sources of pollution, were selected.

### Water sampling and chemical analysis

A one-time, spatially intensive sampling approach was carried out in order to understand the relationship between land use and water quality within headwater catchments of the Upper Vltava watershed. A total of 37 water samples were taken within the low-order streams using conventional sampling protocols. Sample collection took place on 2^nd^ May and 3^rd^ May, 2016 under stable weather conditions. The water sampling locations were used as the catchment outlet for each catchment area in order to consider the land area that supplies water to each sample. Grab samples of electrical conductivity (EC, μS/cm), dissolved oxygen (DO, mg/L) pH and water temperature (TEM, °C) were measured in the field using a portable meter (Hach HQ40d). Laboratory analysis was conducted for the determination of nine WQP, including chemical oxygen demand by dichromate (COD, mg/L), total suspended solids (TSS mg/L), ammonium ions (NH_4_^+^, mg/L), nitrite nitrogen (NO_2_^−^N, mg/L), nitrate nitrogen (NO_3_^−^N, mg/L), total phosphorus (TP, mg/L), orthophosphate phosphorus (PO_4_3^−^P, mg/L), absorbance wavelength 254 (A_254_) and calcium (Ca, mg/L). Storage, preservation and analysis of water samples were conducted according to the standardized methods of the Czech National Standards Criterion^35^. Dissolved oxygen (DO) was omitted from analysis since the majority of streams were highly saturated with oxygen and there were minimal differences in the concentration of DO between streams. Water temperature was also disregarded in the evaluation due to the lack of seasonal influences from the one-time sampling.

### Landscape composition metrics

GIS analysis via ArcMap 10.5.1 (ESRI) was used to acquire land use information. Catchment areas and streams were delineated via shapefiles provided by the Czech Digital Base of Water Management Data^36^. Detailed aerial images from 2015 were supplied by the public ArcGIS Online Map Service from the Czech Office for Surveying and Cadastre (www.cuzk.cz) and were used to determine the land use composition (i.e., the percent of each land use category) in each catchment area at a mapping scale of 1:5000, thus allowing for a precise analysis of the landscape structure. However, an aerial view via www.mapy.cz was used as a reference in case of any discrepancies. All shapefiles and layers were created using the coordinate system S-JTSK Krovak East North. Land use was classified into five categories: (1) settlements, including houses, parking lots and other infrastructure; (2) cropland, including rows of agriculture, cultivated crops and orchards; (3) meadows, including pastures, mowed areas and grass communities; (4) forests, including groups of trees and shrubs: and (5) water bodies including reservoirs, ponds and lakes. All catchments are primarily covered with forests and meadows, while small fragments of croplands, settlements and water bodies occupy less than 4% of the total study area. Incorporating the influence of land use types that are not present in every catchment creates problems with statistical analysis due to many zeros in the dataset, for that reason, croplands, settlements and water bodies were removed from analysis.

Following the approach proposed by Peterson^19^ and Peterson & Pearse^11^, the weighting of land use proportions was based on an arrangement of site-specific explanatory variables, including the inverse Euclidean distance of each raster cell to the stream, the inverse flow length (i.e., the inverse distance to the stream measured along the flow path identified on Digital Elevation Model) and flow accumulation. Additionally, slope was included as a supplementary explanatory variable to augment metrics. It is common practice in hydrology to use log-transformed values of flow accumulation in many applications due to its typical exponential frequency distribution (e.g., topographic wetness index), thus, a logarithmic transformation was also applied to metrics containing flow accumulation data. Using various, multiplicative combinations of these weights, thirteen landscape composition metrics were defined; each containing an inverse-distance function measured with either Euclidean distance or flow length, henceforth referred to as “Euclidean metrics” and “flow metrics”, with the exception of the unweighted metric which only considered land use composition (see Table 1 for the complete list of metrics). Metrics were implemented via a Python script, utilizing the functionality of Spatial Analyst toolbox for ArcGIS 10.5 (ESRI, 2017) via ArcPy module (see Supplementary Method S1). A 5 m resolution Digital Terrain Model of the Czech Republic of the 5th generation (DMR 5G) was provided by the Czech Office for Surveying, Mapping and Cadastre and used to attain raster data for the calculation of slope, flow paths and flow accumulation.

**Table 1:** Variables, abbreviations and descriptions of landscape composition metrics applied to each land use category within a catchment.

Variables	Abbreviation	Description	Equation
None	Unweighted	Percentage of land use; no spatial or topographic considerations	$\% {\rm{LU}}=\frac{{\sum }_{{\rm{i}}=1}^{{\rm{n}}}{{\rm{I}}}_{{\rm{i}}}({\rm{k}})}{{\rm{n}}}\times 100$
Stream proximity	Euclid	Inverse Euclidean distance from land use to tributary	$\% {\rm{LU}}=\frac{{\sum }_{{\rm{i}}=1}^{{\rm{n}}}{{\rm{I}}}_{{\rm{i}}}({\rm{k}}){{\rm{E}}}_{{\rm{i}}}}{{\sum }_{{\rm{i}}=1}^{{\rm{n}}}{{\rm{E}}}_{{\rm{i}}}}\times 100$
Stream proximity, Slope	Euclid-S	Inverse Euclidean distance from land use to tributary and slope degree of land use	$\% {\rm{LU}}=\frac{{\sum }_{{\rm{i}}=1}^{{\rm{n}}}{{\rm{I}}}_{{\rm{i}}}({\rm{k}}){{\rm{E}}}_{{\rm{i}}}{{\rm{S}}}_{{\rm{i}}}}{{\sum }_{{\rm{i}}=1}^{{\rm{n}}}{{\rm{E}}}_{{\rm{i}}}{{\rm{S}}}_{{\rm{i}}}}\times 100$
Stream proximity, Flow Accumulation	Euclid-A	Inverse Euclidean distance from land use to tributary and pathways of flow accumulation within land use	$\% \mathrm{LU}=\frac{{\sum }_{{\rm{i}}=1}^{{\rm{n}}}{{\rm{I}}}_{{\rm{i}}}({\rm{k}}){{\rm{E}}}_{{\rm{i}}}{{\rm{A}}}_{{\rm{i}}}}{{\sum }_{{\rm{i}}=1}^{{\rm{n}}}{{\rm{E}}}_{{\rm{i}}}{{\rm{A}}}_{{\rm{i}}}}\times 100$
Stream proximity, Flow Accumulation	Euclid-LogA	Inverse Euclidean distance from land use to tributary and logarithmically transformed pathways of flow accumulation within land use	$\% \mathrm{LU}=\frac{{\sum }_{i=1}^{{\rm{n}}}{{\rm{I}}}_{{\rm{i}}}({\rm{k}}){{\rm{E}}}_{{\rm{i}}}\,\mathrm{ln}\,({{\rm{A}}}_{{\rm{i}}})}{{\sum }_{i=1}^{{\rm{n}}}{{\rm{E}}}_{{\rm{i}}}\,\mathrm{ln}\,({{\rm{A}}}_{{\rm{i}}})}\times 100$
Stream proximity, Slope, Flow Accumulation	Euclid-SA	Inverse Euclidean distance from land use to tributary, slope degree of land use and flow accumulation within land use	$\% \mathrm{LU}=\frac{{\sum }_{{\rm{i}}=1}^{{\rm{n}}}{{\rm{I}}}_{{\rm{i}}}({\rm{k}}){{\rm{E}}}_{{\rm{i}}}{{\rm{S}}}_{{\rm{i}}}{{\rm{A}}}_{{\rm{i}}}}{{\sum }_{{\rm{i}}=1}^{{\rm{n}}}{{\rm{E}}}_{{\rm{i}}}{{\rm{S}}}_{{\rm{i}}}{{\rm{A}}}_{{\rm{i}}}}\times 100$
Stream proximity, Slope, Flow Accumulation	Euclid-SlogA	Inverse Euclidean distance from land use to tributary, slope degree of land use and logarithmically transformed flow accumulation within land use	$\% {\rm{LU}}=\frac{{\sum }_{{\rm{i}}=1}^{{\rm{n}}}{{\rm{I}}}_{{\rm{i}}}({\rm{k}}){{\rm{E}}}_{{\rm{i}}}{{\rm{S}}}_{{\rm{i}}}\,\mathrm{ln}\,({{\rm{A}}}_{{\rm{i}}})}{{\sum }_{{\rm{i}}=1}^{{\rm{n}}}{{\rm{E}}}_{{\rm{i}}}{{\rm{S}}}_{{\rm{i}}}\,\mathrm{ln}\,({{\rm{A}}}_{{\rm{i}}})}\times 100$
Stream proximity	Flow	Inverse flow length from land use to tributary	$\% {\rm{LU}}=\frac{{\sum }_{{\rm{i}}=1}^{{\rm{n}}}{{\rm{I}}}_{{\rm{i}}}({\rm{k}}){{\rm{F}}}_{{\rm{i}}}}{{\sum }_{{\rm{i}}=1}^{{\rm{n}}}{{\rm{F}}}_{{\rm{i}}}}\times 100$
Stream proximity, Slope	Flow-S	Inverse flow length from land use to tributary and slope degree of land use	$\% {\rm{LU}}=\frac{{\sum }_{{\rm{i}}=1}^{{\rm{n}}}{{\rm{I}}}_{{\rm{i}}}({\rm{k}}){{\rm{F}}}_{{\rm{i}}}{{\rm{S}}}_{{\rm{i}}}}{{\sum }_{{\rm{i}}=1}^{{\rm{n}}}{{\rm{F}}}_{{\rm{i}}}{{\rm{S}}}_{{\rm{i}}}}\times 100$
Stream proximity, Flow Accumulation	Flow-A	Inverse flow length from land use to tributary and pathways of flow accumulation within land use	$\% {\rm{LU}}=\frac{{\sum }_{{\rm{i}}=1}^{{\rm{n}}}{{\rm{I}}}_{{\rm{i}}}({\rm{k}}){{\rm{F}}}_{{\rm{i}}}{{\rm{A}}}_{{\rm{i}}}}{{\sum }_{{\rm{i}}=1}^{{\rm{n}}}{{\rm{F}}}_{{\rm{i}}}{{\rm{A}}}_{{\rm{i}}}}\times 100$
Stream proximity, Flow Accumulation	Flow-logA	Inverse flow length from land use to tributary and logarithmically transformed pathways of flow accumulation within land use	$\% {\rm{LU}}=\frac{{\sum }_{{\rm{i}}=1}^{{\rm{n}}}{{\rm{I}}}_{{\rm{i}}}({\rm{k}}){{\rm{F}}}_{{\rm{i}}}\,\mathrm{ln}\,({{\rm{A}}}_{{\rm{i}}})}{{\sum }_{{\rm{i}}=1}^{{\rm{n}}}{{\rm{F}}}_{{\rm{i}}}\,\mathrm{ln}\,({{\rm{A}}}_{{\rm{i}}})}\times 100$
Stream proximity, Slope, Flow Accumulation	Flow-SA	Inverse flow length from land use to tributary, slope degree of land use and pathways of flow accumulation within land use	$\% {\rm{LU}}=\frac{{\sum }_{{\rm{i}}=1}^{{\rm{n}}}{{\rm{I}}}_{{\rm{i}}}({\rm{k}}){{\rm{F}}}_{{\rm{i}}}{{\rm{S}}}_{{\rm{i}}}{{\rm{A}}}_{{\rm{i}}}}{{\sum }_{{\rm{i}}=1}^{{\rm{n}}}{{\rm{F}}}_{{\rm{i}}}{{\rm{S}}}_{{\rm{i}}}{{\rm{A}}}_{{\rm{i}}}}\times 100$
Stream proximity, Slope, Flow Accumulation	Flow-SlogA	Inverse flow length from land use to tributary, slope degree of land use and logarithmically transformed flow accumulation within land use	$\% {\rm{LU}}=\frac{{\sum }_{{\rm{i}}=1}^{{\rm{n}}}{{\rm{I}}}_{{\rm{i}}}({\rm{k}}){{\rm{F}}}_{{\rm{i}}}{{\rm{S}}}_{{\rm{i}}}\,\mathrm{ln}\,({{\rm{A}}}_{{\rm{i}}})}{{\sum }_{{\rm{i}}=1}^{{\rm{n}}}{{\rm{F}}}_{{\rm{i}}}{{\rm{S}}}_{{\rm{i}}}\,\mathrm{ln}\,({{\rm{A}}}_{{\rm{i}}})}\times 100$

Notes: %LU = Percentage of land use category; n = total number of cells in the catchment; I_i_(k) = presence of land use k in cell i (1 or 0); E_i_ = inverse Euclidean distance from cell i to the stream (distance +1)^−1^; F_i_ = inverse flow length from cell i to the stream (distance +1)^−1^; S_i_ = slope gradient for cell i; A_i_ = flow accumulation value for cell i.

### Statistical analysis

Outliers of COD and A_254_ for sites 5 and 6 were excluded from analysis due to the possibility of riverbank stabilization efforts affecting these parameters during the time of sampling. To investigate the differences between weighting schemes, Pearson’s correlation coefficient analysis was computed for all pairs of landscape composition metrics. A separate linear regression model was then fitted for each combination of WQP (response), landscape composition metric (predictor) and land use category to assess how certain metrics can influence land use predictions of chemical concentrations. The predictive power of the models was then compared using *R*^2^ values. R statistical software (R Core Team 2018) was used for all data manipulation, computation and graphics.

## Results

### Variation between landscape composition metrics

Both land use categories experienced changes in proportions when spatial proximity and topography were incorporated into landscape composition metrics (see Supplementary Tables S2 and S3). The unweighted proportions of forests and meadows within catchments were approximately 65 ± 20 (mean ± SD) and 32 ± 17 (mean ± SD), respectively (Table 2). For both forests and meadows, the Euclidian distance metric (i.e., Euclid) led to similar mean proportions as the land use composition metric (i.e., Unweighted). Employing more complex weighting schemes, however, led to an increase in proportions of forests and a decrease in proportions of meadows, with the change varying from approximately 2 to 8%. Additionally, standard deviations of proportions experienced substantial variations, with Euclidean metrics containing untransformed flow accumulation increasing in standard deviations by approximately 10% (Table 2).

**Table 2:** Mean and standard deviation for proportions of forests and meadows within catchments measured by various landscape composition metrics.

*Metric*	*Forests*		*Meadows*	
	*Mean*	*SD*	*Mean*	*SD*
Unweighted	64.82	19.6	31.6	17.4
Euclid	65.13	24.84	31.61	22.89
Euclid-S	71.55	20.97	26.11	19.43
Euclid-A	71.36	29.81	25.93	27.44
Euclid-logA	68.18	23.19	28.8	21.64
Euclid-SA	71.97	29.53	25.24	27.05
Euclid-SlogA	71.78	21.48	25.92	20.09
Flow	67.06	18.37	29.9	16.75
Flow-S	73.01	16.97	24.82	15.51
Flow-A	66.55	21.99	29.49	19.21
Flow-logA	67.47	18.78	29.56	17.05
Flow-SA	71.54	19.51	25.27	16.44
Flow-SlogA	73.16	17.5	24.71	15.89

**Figure 2:**
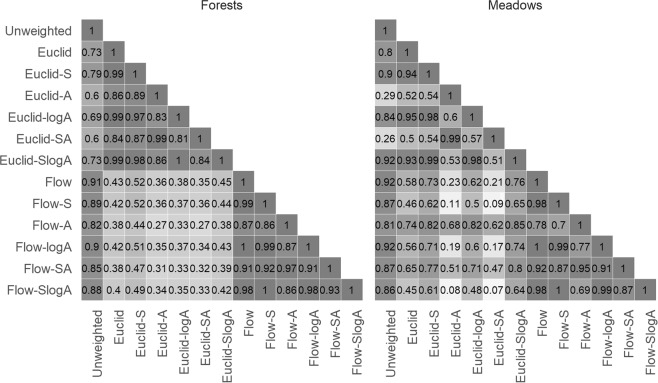
Pearson’s correlation coefficients between pairs of landscape composition metrics for each land use category.

Pair-wise correlations between landscape composition metrics were 0.70 ± 0.27 (mean ± SD) for forests and 0.73 ± 0.26 (mean ± SD) for meadows (Fig. 2). Forests displayed relatively weak correlations between Euclidean and flow metrics (0.39 ± 0.06; see the light-colored rectangular section in the Forests portion of Fig. 2). The weakest correlations for meadows were observed in most pairs that included either Euclid-A or Euclid-SA (0.45 ± 0.28; see the light-colored stripes in the Meadows portion of Fig. 2), which indicates that metrics including inverse Euclidean distance in combination with flow accumulation (that is not logarithmically transformed) are the least similar to other metrics. The highest correlations were observed for pairs consisting of any flow metric when compared to the same metric enriched by log-transformed flow accumulation (0.99 ± 0.02), as well as for pairs of any Euclidean metric without slope compared to the same metric with slope (0.98 ± 0.02), indicating that the log-transformation of flow metrics as well as the addition of slope for Euclidean metrics results in limited changes of weighted proportions.

### Landscape composition metric for predictions of water quality

For parameters A_254_, NH_4_^+^, and COD, no significant relationships were found between chemical concentrations and proportions of forests or meadows, regardless of which metric was applied (see Supplementary Table S4); hence, the results for these three parameters are not shown and disregarded from further analysis. Overall, the percentage of explained water quality variance ranged from 1 to 46% for forests and from 0.1 to 32% for meadows. The water quality variability principally followed the same pattern for both land use categories, albeit with lower R^2^ values for meadows in all cases; consequently, only the results obtained from forests as predictors of water quality are discussed.

**Figure 3:**
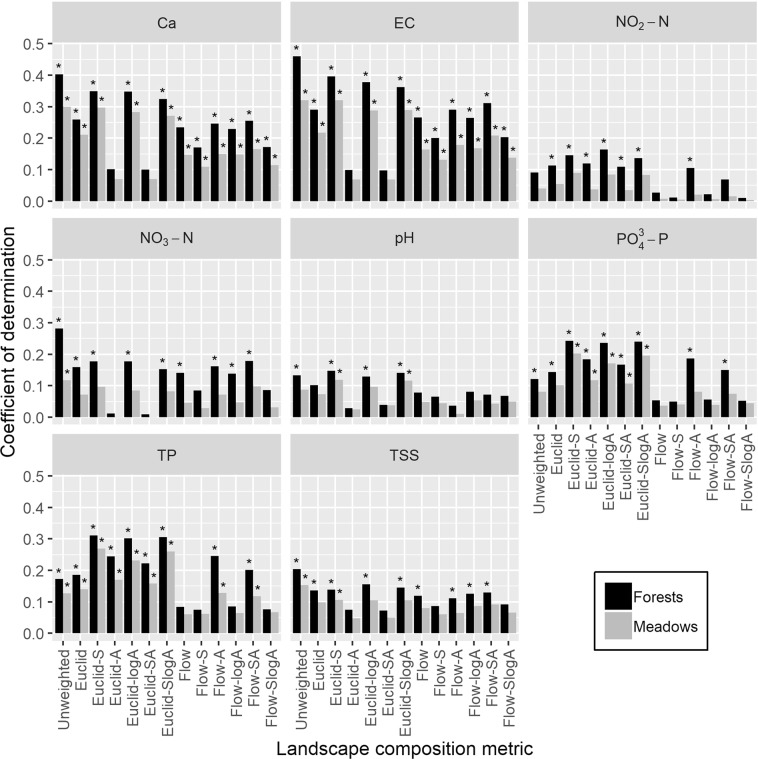
Coefficients of determination (*R*^2^) for linear regressions of water quality parameters (WQP) for proportions of forests and meadows. A separate linear model was fitted for each combination of land use category, WQP, and landscape composition metric. The significant models are denoted with an asterisk.

There were substantial variations in performance between the landscape composition metrics for most WQP, often leading to differences in their significance (see Fig. 3 for comparison of coefficients of determination and Supplementary Table S4 for regression slopes and their standard errors). The unweighted metric and Euclidean metrics frequently exceeded corresponding flow metrics in explained variations of water quality parameters. An exception was with Euclidean metrics employing an untransformed flow accumulation variable (i.e., Euclid-A and Euclid-SA) which created inferior predictions for parameters Ca, EC, NO_3_^−^N, pH and TSS. The *R*^2^ values for models incorporating Euclidean distance in combination with slope (i.e., Euclid-S), log-transformed flow accumulation (i.e., Euclid-logA) or both slope and log-transformed flow accumulation (i.e., Euclid-SlogA) were relatively similar, typically ranging in approximately 5%, with moderately lower R^2^ values for most models when including only Euclidean distance (i.e., Euclid).

Two slightly different versions of a similar pattern in variation can be identified among parameters in Fig. 2. For Ca, EC, NO_3_^−^N, and TSS, the best prediction was obtained by the basic, unweighted proportions, followed by Euclid-S, Euclid-logA, Euclid-SlogA, yielding approximately 10% lower R^2^ values. For the remaining WQP, Euclid-S, Euclid-logA, Euclid-SlogA resulted in the highest R^2^ values, whereas the performance of unweighted metric was either similar (as seen in predictions of pH) or approximately 10 to 20% lower (as seen in predictions of NO_2_^−^N, PO_4_^3−^P and TP).

## Discussion

The effects of land use on concentrations of specific WQP were investigated at the catchment scale using landscape composition metrics, a broadly-applicable weighting scheme that considers the combined effects of individual land use categories with spatial and topographical variables. As in previous studies^10,16,17^, two conventional measures used in ArcGIS for calculating distance were employed (i.e., flow length and Euclidean distance), as well as a non-spatial composition measurement (i.e., unweighted metric), to compare the predictive ability between metric types. Both King^10^ and Peterson^19^ found that Euclidean distance and flow length metrics were very strongly correlated with one another. On the contrary, disparity was found between relationships and performance of flow length and Euclidean distance in our study, particularly for parameters NO_2_-N, pH, PO_4_^3^-P and TP. Results from the regression models revealed that metrics with a Euclidean distance measure predominately outperformed metrics containing a flow length measure. The weaker performance of flow metrics in our study was most likely due to the artificial flow paths parallel to the stream. Such parallel flow paths are a well-known feature of the Single Flow with 8 Directions (SFD8) algorithm^37^, which is the most commonly used and often the only implemented algorithm in ArcGIS for the determination of outflow from a Digital Terrain Model cell. However, when flow length distance was combined with flow accumulation (i.e., Flow-A or Flow-SA), the predictive power for forests comparatively improved for certain WQP. Presumably, the low weights produced by artificially long flow paths are compensated by the higher flow accumulation downstream, since flow can accumulate to a greater extent along lengthy flow paths. Thus, caution should be taken when implementing flow length as a distance measure when flow accumulation is not being considered. Since any distance function can be employed into metrics^19^, future research should explore alternative flow direction algorithms which may be more accurate in displaying near-stream flow pathways, although it is not clear how to define a flow-based distance in the presence of flow divergence. Given these findings, metrics which implemented a flow length measure were removed from further discussion.

This study follows the methodology proposed by Peterson^19^ and is further augmented to examine how applying a log-transformation function for flow accumulation variables, as well as integrating a slope variable, can influence metric performance. When stream proximity, calculated with a Euclidean distance measure, was combined with untransformed flow accumulation (i.e., Euclid-A and Euclid-SA), R^2^ values were often drastically altered, frequently diminishing significant predictions for certain WQP. Hence, there seems to be no justification for including flow accumulation that is not log-transformed into landscape composition metrics when predicting stream water quality. Moreover, the differences in performance between metrics incorporating slope gradient (i.e., Euclid-S), log-transformed flow accumulation (i.e., Euclid-logA), or both slope and log-transformed flow accumulation (i.e., Euclid-SlogA) were minor. This suggests that slope and log-transformed flow accumulation produced extremely similar effects and that the inclusion of both physiographic attributes adds unnecessary complexity and is not vital for enhancing water quality predictions. However, when compared to slope gradient, flow accumulation is relatively difficult to compute, hence Euclid-S may be the more straightforward option for metric implementation.

Both the spatial proximity^10,17,19,38,39^ and the topography^2,9,39–42^ of land use are regarded as crucial factors influencing stream condition. However, the inclusion of stream proximity, exclusively, never resulted in optimal water quality predictions (Fig. 3). Previous studies have suggested that the predictive ability of metrics may be connected to the size of the catchment or watershed^10,14,19^ and the fact that the examined catchments within our study area were small in size (average catchment area ~6 km²) may be a contributing factor to this presumed effect. Within a small spatial extent, most land use can have direct pathways of influence^2,43^ and, therefore, an inverse distance measure may be negligible when examining small catchments. On the other hand, when stream proximity was combined with slope (i.e., Euclid-S), log-transformed flow accumulation (i.e., Euclid-logA) or a combination of both (i.e., Euclid-SlogA), the explained variability in water quality data often increased, confirming the importance of landform. Albeit small in surface area, the hilly, submontane terrain of this region may account for the more accurate predictions produced when slope and log-transformed flow accumulation were considered, thus, incorporating topographic variables into metrics may be pivotal for submontane regions. The influence of landscape features could be more significant when human activity is limited^2^, which is the case within our sparsely populated study area. Nevertheless, these findings are circumstantial and should not be taken out of context; the influence of stream proximity and topography could further increase with larger catchment sizes^2,10^; thus, the extent to which these factors have an influence requires further investigation.

While accounting for both spatial and topographic attributes improved the predictive ability of models for parameters of pH, TP, NO_2_^−^N and PO_4_3^−^P, the incorporation of stream proximity, slope and flow accumulation did not always explain the most variability in water quality data (Fig. 3). Both unweighted metrics and Euclidean metrics were optimal for predicting chemical loading, depending on which WQP was being considered. This behavior is conceivably due to the regional processes and mechanisms which govern these parameters^14^ and suggests that the topography and spatial proximity of land use did not have an impact on the conveyance of Ca, EC, NO_3_^−^N and TSS, yet that land use composition was a dominant factor impacting these parameters. The dissimilar pattern found between these two groups of parameters can be attributed to the different geochemical cycles which can react conversely; Ca, EC, NO_3_^−^N and TSS are relatively stable parameters, whereas pH, TP, NO_2_^−^N and PO_4_^3−^P are typically reactive or unstable^44^, especially in oligotrophic waters with very low concentrations which may produce highly variable ratios^45^. Hence, the factors governing land use-water quality interactions could be contingent on the reactiveness and stability of individual WQP. However, water quality can be influenced by multiple sources of contamination through dynamic pathways and at various scales and thorough information on the interactions between different nutrients and their mechanistic processes is lacking^46^. Consequently, no particular metric should be used to predict the chemical concentrations of every parameter. With the application of multiple landscape composition metrics, the relationship between land use and water quality can be examined according to the most appropriate metric which explains the highest variability in data. However, this should not lead to automatic post-hoc methodological decisions based on a limited sample size, as the outperformance of certain metrics could just be a matter of chance. One should always have a sound theoretical justification why specific metrics should be preferred over others. For instance, an inverse distance measure may be more influential in large catchments, whereas flow accumulation and slope gradient might be less significant in regions with flat terrains; hence, potential factors such as catchment size and topography should be considered when inferring metrics performance.

The protection of freshwater resources and ecosystems requires an understanding of the impacts from the surrounding land use, yet, determining the optimal spatial extent for examining land use-water quality relationships, as well as accounting for landscape attributes and processes, are currently ongoing issues facing researchers. Since each land use can impose a varying degree of influence on water quality, weighting specific land use categories according to spatial proximity and topography is an efficient way to account for the contributing, scale dependent responses and mechanisms throughout a landscape^3,13^. Still, it should not be assumed that stream proximity, slope gradient and flow accumulation are the only variables impacting land use-water quality interactions. Recently, studies have concluded that landscape patterns^39,40,47–49^ and soil type^42,46,50–53^ can also impact water quality; therefore, it may be beneficial for future studies to include additional variables, such as patch size of landscape elements or soil properties. Landscape composition metrics are easily reproducible approaches that have seldom been implemented and explored. The ability of weighting-schemes to integrate multiple variables creates an opportunity for further advancement of land use-water quality assessment and the potential for more accurate predictive models.

This work addresses the prevalent land use categories within the study area: forests and meadows. Inevitably, the proportions of forests and meadows were significantly correlated for all applied metrics (see Supplementary Table S5), with an overall mean correlation coefficient value (±SD) of −0.97 (±0.01), resulting in both land use categories having similar optimal metrics for each WQP. Therefore, it is unknown how other land use categories would respond to the applied metrics. King^10^ found that an adequate range of land use percentage is necessary to avoid hindering the performance of certain metrics. The catchments within our study area contain 3.5–79.9% of meadows and 14.6–96.5% of forests, accounting for varying extents, while the proportions of croplands and settlements represent only small percentages within catchments, making computation problematic. Thus, their influence should be captured in another way than by percent composition.

Due to the one-time sample collection, the results represent water quality from a single point in time. However, rainfall, temperature and land use activities change depending on season, creating variations in flow rates, surface runoff and contaminant input to receiving waters^38,40,47,54,55^. Hence, seasonal effects should be incorporated whenever time series data is available.

## Conclusions

Herein, landscape composition metrics were employed to discern the relative significance of stream proximity, slope and flow accumulation on predictions of water quality within headwater catchments via the incorporation of alternate spatial measures, functions and landscape variables. Overall, there were significant variations in performance between the landscape composition metrics; land use composition (i.e., unweighted metric) and stream proximity measured with Euclidean distance (i.e., Euclidean metrics) predominantly outperformed stream proximity measured with flow length (i.e., flow metrics) in predicting most land use-water quality relationships. Incorporating slope or a logarithmic transformation of flow accumulation in combination with a Euclidean distance measure of stream proximity (i.e., Euclid-S or Euclid-logA) often improved model accuracy, yet integrating both topographic variables (i.e., Euclid-SlogA) never resulted in optimal predictions. Euclid-S or Euclid-logA explained the highest variability in pH, TP, NO_2_-N and PO_4_^3^-P, while the unweighted metric was most effective for predicting concentrations of Ca, EC, NO_3_-N and TSS. The results suggest that the spatial position and terrain of land use can govern the conveyance of reactive or unstable water quality parameters, whereas the proportions of land use are dominant factors for predicting more stable chemical data. Thus, the application of the unweighted metric as well as the Euclid-S or Euclid-logA metric is recommended for optimal model accuracy when examining the effects of land use on water quality in small, submontane catchments. With the implementation of landscape composition metrics, management efforts can be directed according to the parameter of concern and the associated, governing processes.

## Supplementary information

Supplementary Information

## Data Availability

The datasets generated during and analyzed during the current study are available in the Mendeley repository, 10.17632/cf5yxs28cv.2.
