# Supplementary material for: Performance of landscape composition metrics for predicting water quality in headwater catchments
Source: Sci Rep. 2019 Oct 8;9:14405. doi: 10.1038/s41598-019-50895-6 (PMC6783472; doi:10.1038/s41598-019-50895-6)
Supplement: Supplementary file 1 — Supplementary Information [file 41598_2019_50895_MOESM1_ESM.pdf]

Performance of landscape composition metrics for predicting water quality in headwater catchments

Linda R. Staponites; Vojtěch Barták; Michal Bílý; Ondřej P. Simon

Supplementary Information

Supplementary Method S1. Python code for computation of landscape composition metrics. Requires ArcGIS 10.1 and Python 2.7 installed.

```
## Importing required modules ---------------------------------------------------------------
import arcpy, numpy, math, os.path

## Reading the input parameters -------------------------------------------------------------
dtm = arcpy.GetParameterAsText(0)      # Path to the digital terrain model
stream = arcpy.GetParameterAsText(1)   # Path to the stream polyline shapefile
mask = arcpy.GetParameterAsText(2)     # Path to the catchment boundary polygon shapefile
landuse = arcpy.GetParameterAsText(3)  # Path to the land use polygon shapefile
field = arcpy.GetParameterAsText(4)    # Name of the land use field in the "landuse" shapefile
output = arcpy.GetParameterAsText(5)   # Path to the output .dbf table
euclid = arcpy.GetParameterAsText(6)   # Boolean indicator whether to use Euclidian or flow
distance

## Dtm extraction ---------------------------------------------------------------------------
arcpy.AddMessage("Extracting dtm...")
dtm = arcpy.sa.ExtractByMask(dtm, mask)

## Enviroments settings ---------------------------------------------------------------------
arcpy.env.cellSize = dtm
arcpy.env.extent = dtm
arcpy.env.mask = mask

## Computing weights ------------------------------------------------------------------------
w = []
arcpy.AddMessage("Computing distance weights...")
if euclid == "true":
    w.append(1/(arcpy.sa.EucDistance(stream)+1))           # w1: Euclidian distance
else:
    fill = arcpy.sa.Fill(dtm)
    dirs = arcpy.sa.FlowDirection(fill)
    strmras = arcpy.FeatureToRaster_conversion(stream, "FID",
os.path.join(os.path.dirname(output),"strmras"))
    rivers = arcpy.sa.BooleanNot(arcpy.sa.IsNull(strmras))
    dirs = arcpy.sa.Con(rivers, 5, dirs)
    w.append(arcpy.sa.FlowLength(dirs, "DOWNSTREAM"))      # w1: stream distance

arcpy.AddMessage("Computing slope weights...")
w.append(w[0]*arcpy.sa.Slope(dtm))                         # w2: distance and slope

arcpy.AddMessage("Computing flow accumulation weights...")
if euclid:
    fill = arcpy.sa.Fill(dtm)
    dirs = arcpy.sa.FlowDirection(fill)
accum = arcpy.sa.FlowAccumulation(dirs)
w.append(w[0]*accum)                               # w3: distance and accumulation
w.append(w[1]*accum)                               # w4: distance and slope and accumulation
laccum = arcpy.sa.Log10(accum)
w.append(w[0]*laccum)                              # w5: distance and log accumulation
w.append(w[1]*laccum)                              # w6: distance and slope and log
accumulation

arcpy.AddMessage("Normalizing weights...")
for i in range(len(w)):
    r_array = arcpy.RasterToNumPyArray(w[i], nodata_to_value = 0)
    sum_r = float(r_array.sum())
    w[i] /= sum_r

## Computing proportions --------------------------------------------------------------------
arcpy.AddMessage("Computing proportions...")
folder = os.path.dirname(output)
arcpy.sa.ZonalStatisticsAsTable(landuse,field,w[0],output,"","SUM")
arcpy.sa.ZonalStatisticsAsTable(landuse,field,w[1],os.path.join(folder,"slope.dbf"),"","SUM")
```

```
arcpy.sa.ZonalStatisticsAsTable(landuse,field,w[2],os.path.join(folder,"accum.dbf"),"","SUM")
arcpy.sa.ZonalStatisticsAsTable(landuse,field,w[3],os.path.join(folder,"slp_ac.dbf"),"","SUM")
arcpy.sa.ZonalStatisticsAsTable(landuse,field,w[4],os.path.join(folder,"log_ac.dbf"),"","SUM")
arcpy.sa.ZonalStatisticsAsTable(landuse,field,w[5],os.path.join(folder,"slp_la.dbf"),"","SUM")
arcpy.JoinField_management(output, field, os.path.join(folder,"slope.dbf"), field, ["SUM"])
arcpy.JoinField_management(output, field, os.path.join(folder,"accum.dbf"), field, ["SUM"])
arcpy.JoinField_management(output, field, os.path.join(folder,"slp_ac.dbf"), field, ["SUM"])
arcpy.JoinField_management(output, field, os.path.join(folder,"log_ac.dbf"), field, ["SUM"])
arcpy.JoinField_management(output, field, os.path.join(folder,"slp_la.dbf"), field, ["SUM"])

## Cleaning up ---------------------------------------------------------------------------
arcpy.Delete_management(os.path.join(folder,"slope.dbf"))
arcpy.Delete_management(os.path.join(folder,"accum.dbf"))
arcpy.Delete_management(os.path.join(folder,"slp_ac.dbf"))
arcpy.Delete_management(os.path.join(folder,"log_ac.dbf"))
arcpy.Delete_management(os.path.join(folder,"slp_la.dbf"))
if euclid != "true":
    arcpy.Delete_management(os.path.join(folder,"strmras"))
```

Supplementary Table S2. Percent of meadows for each landscape composition metric (horizontal axis) of each catchment (vertical axis).

Sample #	%	Flow	Flow-S	Flow-A	Flow-SA	Flow-logA	Flow-SlogA	Euclid	Euclid-S	Euclid-A	Euclid-SA	Euclid-LogA	Euclid-SlogA
1	11.6	11.7	9.3	11.1	9.1	11.5	9.3	7	6	1	1	6.4	5.7
2	9	8.8	6.7	21	10.9	8.3	6.4	10.2	6.1	17.3	10.2	11.3	6.3
5	20.6	17.3	11.1	17.5	13.6	15	9.8	27	23.1	20.1	18.2	24.5	21.3
6	34	27.4	20.1	66.5	39.7	26.6	19.2	46.1	42	59.3	53.6	44.9	40.1
10	5.4	4.7	5	2.7	4.3	4.3	4.6	3.7	3.9	1	0.9	3.4	3.6
15	3.5	1.9	1.6	3.1	1.1	1.4	1.3	6.7	2.7	7.6	4.3	4.6	2
17	14	9.8	6.7	6.8	6.2	9	6.2	11.9	9	4.5	5.7	10.5	8
18	33.8	23.5	16	40.6	23.6	21.4	14.5	51.6	41.2	53.2	47.9	52.8	42.2
19	34.8	31	26.7	40.7	39.2	29.9	25.7	36.1	36.1	7.6	5.4	31.7	32.2
20	51.3	46.8	35.6	39.3	29.5	44.5	33.9	31.3	23.5	14.3	9.1	27.5	21
21	33.8	24.6	18.5	19.1	17.3	23.6	17.8	23.5	19.8	4.2	3	19.6	17.1
22	22.1	21.6	15	21.5	19.1	21.7	14.9	19.8	18.8	21.7	42.5	18.2	18.5
23	28.3	23.2	18.4	21.5	21.1	22.9	18.4	30.4	28	24.8	26.1	29.8	28.1
24	32.1	29	19.7	26.4	20.8	26.4	17.9	26.5	20.2	10.4	9.7	23.3	18.5
25	31.7	27.5	21.8	29.2	30.8	28.8	23.2	19.9	21.7	8.3	9.6	18.6	21
27	44.2	43	32.2	50.5	42.8	43	32.1	46.2	36.7	17.9	17.9	44.4	35.2
28	79.7	71.3	65.9	63.2	56.9	72.4	67.6	96.1	92.4	99.8	99.7	96.8	93.7
30	30.5	27.2	20.8	24.9	19.8	28	21.4	24.9	20.3	6.5	6.9	24.3	20.5
33	18.2	21.5	15.8	7.9	8.1	20.2	14.9	16.4	11.1	5.8	4.1	16.1	10.9
35	33	27.7	23.3	39.7	32.5	27.3	23.1	35.3	32.2	27.7	20.1	35.9	31.8
38	20.3	22.6	17.5	11.9	11.1	21.6	17	12.2	9.5	1.1	1.3	10.9	8.9
42	22.1	24.7	19.2	17.4	13.3	25	19.5	31.4	27.9	55.5	62.7	33.8	30.8

47	31.4	34.4	32.7	16.1	22.8	33.5	32	21.2	25.9	18.3	19.2	20.8	26.5
48	24.3	24.6	20.2	24.7	21.7	24.3	20	27.2	24.3	29.5	31	28.2	25.2
50	26.5	28.5	23	29.7	20.8	29.1	23.6	17.3	16.1	9.6	9.1	16.7	15.6
51	62.6	60.3	47.2	68.6	60.6	60.7	47.7	54.8	53.9	66.8	61.4	57.9	56.3
54	21.4	24.8	20.6	16.9	14.4	25	20.5	16.7	16.8	26.4	28.1	16.9	17
55	48.6	40.9	36.9	42.1	38.4	41.8	38	61.1	54.2	81.3	72.7	63.8	55.9
56	48.4	52	51.4	42.9	44.8	53.1	52.83	44	40.9	70.6	69.8	46.1	42.6
57	22.6	28.3	24.7	16.8	17.7	28.3	25	7.2	7.6	2	2.2	6.1	6.9
58	21.2	33	30	15	13.2	29.7	27.4	6.6	5.8	0.9	0.6	5.1	4.6
59	52.4	68	61.2	49.6	44.4	65.4	58.9	19.4	15	2.3	1.9	17.5	13.9
65	55.4	47.7	42.1	49.2	41.5	49.3	43.8	58.6	51.4	78.4	77.5	63	56.2
67	13.2	10.6	8.5	5.8	5.6	10.6	8.5	8.1	7.6	0.6	0.9	7.3	7.3
68	16	10.9	8.3	10.9	9.6	11.4	8.6	9.2	8.3	4.7	3.6	8.4	8.1
70	50.3	42.3	38.8	64.5	56.3	42.7	39.7	62.2	56.2	53.5	52.3	60.6	55.7
71	60.9	53.1	45.9	55.8	52.3	56	48.9	58.5	49.9	44.9	43.8	58	49.9

Note: see Table 1 in manuscript for a complete description of each landscape composition metric.

Supplementary Table S3. Percent of forests for each landscape composition metric (horizontal axis) of each catchment (vertical axis).

Sample #	%	Flow	Flow-S	Flow-A	Flow-SA	Flow-logA	Flow-SlogA	Euclid	Euclid-S	Euclid-A	Euclid-SA	Euclid-LogA	Euclid-SlogA
1	86.5	86.6	89.7	80.7	86.6	87	89.9	92.1	93.4	98.9	98.9	92.8	93.8
2	90	90.5	92.9	77.9	88.5	91	93.3	88.8	93.5	80.4	85.6	86.8	93
5	78.8	81.6	88.3	81.7	85.9	84.2	89.7	72.9	76.8	79.7	81.7	75.4	78.6
6	64.7	71.5	79	32.4	59.4	72.2	80	52.4	57	39	44.8	53	58.7
10	94.6	95.3	95	97.3	95.7	95.7	95.4	96.2	96.1	99	99.1	96.6	96.4
15	96.5	98.1	98.4	96.9	98.9	98.6	98.7	93.3	97.3	92.4	95.7	95.4	98
17	86	90.2	93.3	93.2	93.8	91	93.8	88.1	91	95.5	94.3	89.5	92
18	57.8	69.5	81.1	44.1	65.9	73.1	83.3	35.4	48.7	18.5	22.7	35.5	49.7
19	50.3	62.5	69.5	47.1	53.4	63.6	70.7	45.7	51.5	91.2	94.2	52.8	57.1
20	48.7	53.2	64.4	60.7	70.5	55.5	66.1	68.7	76.5	85.7	90.9	72.5	79
21	66.2	75.4	81.5	80.9	82.7	76.4	82.2	76.4	80.1	95.8	97	80.4	82.9
22	76.1	75.9	83.4	71.3	78.5	76	83.7	78.2	80.3	76.8	57.3	78.6	80.7
23	58.8	68.2	76.8	55.2	63.4	68.3	76.7	54.3	61.7	49.1	50.8	53.7	60.6
24	60.8	65	77.4	69.8	76.5	68.4	79.6	67.8	75.6	84	83.7	70.1	76.6
25	52.5	65.7	74.1	67	66.3	64.3	72.7	64.2	68	91.2	90.1	68.4	69.9

27	55.2	56.6	67.5	49.4	57.1	56.7	67.7	53.3	62.7	82.1	82.1	55.3	64.3
28	14.6	22.4	28.6	22.3	28.2	21.7	27	2.7	5.9	0.1	0.2	2.2	4.9
30	66.8	71.5	78.5	72.9	79	70.9	77.8	69.2	75.2	90.9	90.9	67.6	72.8
33	77	73.4	81.6	89.5	90	75.1	82.8	82.3	88	94.2	95.9	82.8	88.3
35	51.7	55.2	61.3	49.7	56.2	55.8	61.6	58.9	62.2	71.2	79.3	58.2	62.8
38	79.3	77.2	82.4	88	88.9	78.2	82.9	87.6	90.4	98.9	98.7	88.9	91
42	76.9	73.3	79.5	78.8	83.7	73.1	79.3	67.6	70.8	43.4	36.2	65.2	68.2
47	68.7	65.6	67.3	83.9	77.2	66.4	68	78.8	74.1	81.7	80.8	79.2	73.5
48	75.7	75.4	79.8	75.3	78.3	75.7	80	72.8	75.7	70.5	69	71.8	74.8
50	73.4	71.4	76.9	70.3	79.1	70.8	76.2	82.7	83.9	90.4	90.9	83.3	84.4
51	35.9	38.2	51.3	29.9	37.1	37.8	50.9	44.9	45.8	33.2	38.6	41.8	43.5
54	78.6	75.2	79.4	83.1	85.6	75	79.5	83.3	83.2	73.6	71.9	83.1	83
55	51.5	59.1	63.1	57.9	61.6	58.2	62	38.8	45.8	18.7	27.3	36.2	44.1
56	50.6	46.4	46.7	56	53.8	45.1	45.3	55.6	58.6	29.3	30.1	53.6	57
57	76.6	71	74.8	82.9	82.1	71.2	74.8	92.6	92.2	98	97.8	93.8	93
58	78.8	67	70	85	86.8	70.3	72.6	93.4	94.2	99.1	99.4	94.9	95.4
59	47.6	32	38.8	50.3	55.5	34.6	41.1	80.6	85	97.6	98	82.5	86.1
65	41.1	49.6	55.8	48.5	56.2	48.2	54.2	36.8	44.1	8	7.8	31.7	38.7
67	86.1	89	91.2	92.5	93.9	89	91.3	91.4	92.2	99.3	99.1	92.2	92.5
68	83.7	88.9	91.5	88.8	90.3	88.4	91.3	90.5	91.6	95.2	96.3	91.4	91.9
70	27	31.2	40	15.9	21.9	28.9	37.4	28	34.3	46.2	47.3	30.7	35.1
71	33.3	42.6	50.4	35.3	38.4	39.9	47.5	34.7	44.1	41.5	38.4	34.8	43.4

Note: see Table 1 in manuscript for a complete description of each landscape composition metric.

Supplementary Table S4. Regression slopes, their standard errors, and R^2 values for regression models of water chemistry parameters on forests/meadows percentage measured by different landscape composition metrics.

		Forests				*Meadows*			
WCP	*Metric*	*Slope*	*SE*	*R^2*	*Sign.*[a]	*Slope*	*SE*	*R^2*	*Sign.*
EC	Unweighted	**-3.0547**	**0.5598**	**0.460**	***	**2.8739**	**0.7069**	**0.321**	***
	Euclid	**-1.9152**	**0.5061**	**0.290**	***	**1.7968**	**0.5770**	**0.217**	**
	Euclid-S	**-2.6480**	**0.5534**	**0.395**	***	**2.5695**	**0.6337**	**0.320**	***
	Euclid-A	-0.9301	0.4754	0.099		0.8457	0.5248	0.069	
	Euclid-logA	**-2.3382**	**0.5079**	**0.377**	***	**2.1892**	**0.5821**	**0.288**	***
	Euclid-SA	-0.9329	0.4802	0.097		0.8575	0.5323	0.069	
	Euclid-SlogA	**-2.4732**	**0.5551**	**0.362**	***	**2.3633**	**0.6265**	**0.289**	***

	Flow	**-2.4754**	**0.6963**	**0.265**	**	**2.1289**	**0.8149**	**0.163**	*
	Flow-S	**-2.3261**	**0.7864**	**0.200**	**	**2.0621**	**0.8968**	**0.131**	*
	Flow-A	**-2.1652**	**0.5717**	**0.291**	***	**1.9409**	**0.7042**	**0.178**	**
	Flow-logA	**-2.4131**	**0.6819**	**0.264**	**	**2.1197**	**0.7987**	**0.168**	*
	Flow-SA	**-2.5246**	**0.6350**	**0.311**	***	**2.4466**	**0.8082**	**0.208**	**
	Flow-SlogA	**-2.2750**	**0.7610**	**0.203**	**	**2.0621**	**0.8719**	**0.138**	*
pH	Unweighted	**-0.0120**	**0.0052**	**0.133**	*	0.0110	0.0060	0.087	
	Euclid	-0.0083	0.0042	0.101		0.0076	0.0046	0.073	
	Euclid-S	**-0.0119**	**0.0048**	**0.148**	*	**0.0115**	**0.0053**	**0.119**	*
	Euclid-A	-0.0037	0.0036	0.028		0.0037	0.0039	0.024	
	Euclid-logA	**-0.0100**	**0.0044**	**0.129**	*	0.0093	0.0048	0.097	
	Euclid-SA	-0.0043	0.0036	0.039		0.0046	0.0040	0.038	
	Euclid-SlogA	**-0.0113**	**0.0047**	**0.141**	*	**0.0110**	**0.0051**	**0.116**	*
	Flow	-0.0098	0.0057	0.078		0.0085	0.0064	0.048	
	Flow-S	-0.0098	0.0062	0.065		0.0088	0.0069	0.045	
	Flow-A	-0.0056	0.0049	0.036		0.0034	0.0057	0.010	
	Flow-logA	-0.0098	0.0056	0.081		0.0088	0.0062	0.053	
	Flow-SA	-0.0089	0.0054	0.072		0.0081	0.0065	0.042	
	Flow-SlogA	-0.0096	0.0060	0.068		0.0090	0.0067	0.049	
COD	Unweighted	0.0003	0.0554	0.000		0.0247	0.0621	0.005	
	Euclid	0.0588	0.0424	0.055		-0.0514	0.0465	0.036	
	Euclid-S	0.0116	0.0517	0.002		0.0019	0.0560	0.000	
	Euclid-A	-0.0113	0.0367	0.003		0.0225	0.0399	0.010	
	Euclid-logA	0.0160	0.0467	0.004		-0.0042	0.0502	0.000	
	Euclid-SA	-0.0061	0.0370	0.001		0.0162	0.0404	0.005	
	Euclid-SlogA	0.0064	0.0505	0.000		0.0060	0.0540	0.000	
	Flow	-0.0402	0.0588	0.014		0.0704	0.0637	0.036	
	Flow-S	-0.0562	0.0636	0.023		0.0838	0.0688	0.043	
	Flow-A	-0.0140	0.0510	0.002		0.0442	0.0591	0.017	
	Flow-logA	-0.0380	0.0577	0.013		0.0663	0.0628	0.033	
	Flow-SA	-0.0193	0.0558	0.004		0.0478	0.0662	0.016	
	Flow-SlogA	-0.0526	0.0618	0.021		0.0789	0.0674	0.040	
TSS	Unweighted	**-0.0572**	**0.0191**	**0.204**	**	**0.0558**	**0.0222**	**0.153**	*
	Euclid	**-0.0368**	**0.0157**	**0.136**	*	0.0340	0.0174	0.099	
	Euclid-S	**-0.0440**	**0.0186**	**0.138**	*	**0.0417**	**0.0204**	**0.107**	*

	Euclid-A	-0.0228	0.0135	0.075		0.0197	0.0149	0.048
	Euclid-logA	**-0.0422**	**0.0166**	**0.156**	*	0.0371	0.0183	0.105
	Euclid-SA	-0.0225	0.0137	0.072		0.0205	0.0151	0.050
	Euclid-SlogA	**-0.0440**	**0.0181**	**0.145**	*	0.0399	0.0198	0.104
	Flow	**-0.0467**	**0.0214**	**0.119**	*	0.0420	0.0240	0.081
	Flow-S	-0.0430	0.0236	0.087		0.0393	0.0262	0.060
	Flow-A	**-0.0376**	**0.0180**	**0.111**	*	0.0327	0.0211	0.064
	Flow-logA	**-0.0468**	**0.0209**	**0.126**	*	0.0428	0.0235	0.086
	Flow-SA	**-0.0458**	**0.0201**	**0.129**	*	0.0456	0.0243	0.091
	Flow-SlogA	-0.0430	0.0228	0.092		0.0400	0.0255	0.066
NH4	Unweighted	-0.0002	0.0004	0.009		0.0000	0.0004	0.000
	Euclid	-0.0002	0.0003	0.010		0.0000	0.0003	0.000
	Euclid-S	-0.0003	0.0004	0.016		0.0001	0.0004	0.004
	Euclid-A	-0.0002	0.0003	0.024		0.0000	0.0003	0.000
	Euclid-logA	-0.0003	0.0003	0.025		0.0001	0.0004	0.003
	Euclid-SA	-0.0002	0.0003	0.020		0.0000	0.0003	0.001
	Euclid-SlogA	-0.0003	0.0004	0.018		0.0001	0.0004	0.004
	Flow	0.0000	0.0004	0.000		-0.0001	0.0005	0.001
	Flow-S	0.0001	0.0005	0.000		-0.0001	0.0005	0.002
	Flow-A	-0.0003	0.0004	0.018		0.0000	0.0004	0.000
	Flow-logA	0.0000	0.0004	0.000		-0.0001	0.0005	0.001
	Flow-SA	-0.0002	0.0004	0.011		0.0000	0.0005	0.000
	Flow-SlogA	0.0000	0.0004	0.000		-0.0001	0.0005	0.002
NO2	Unweighted	-0.0001	0.0001	0.091		0.0001	0.0001	0.040
	Euclid	**-0.0001**	**0.0000**	**0.113**	*	0.0001	0.0000	0.054
	Euclid-S	**-0.0001**	**0.0001**	**0.145**	*	0.0001	0.0001	0.089
	Euclid-A	**-0.0001**	**0.0000**	**0.120**	*	0.0000	0.0000	0.037
	Euclid-logA	**-0.0001**	**0.0000**	**0.164**	*	0.0001	0.0001	0.084
	Euclid-SA	**-0.0001**	**0.0000**	**0.109**	*	0.0000	0.0000	0.035
	Euclid-SlogA	**-0.0001**	**0.0000**	**0.137**	*	0.0001	0.0001	0.083
	Flow	-0.0001	0.0001	0.026		0.0000	0.0001	0.007
	Flow-S	0.0000	0.0001	0.011		0.0000	0.0001	0.004
	Flow-A	**-0.0001**	**0.0000**	**0.106**	*	0.0000	0.0001	0.020
	Flow-logA	-0.0001	0.0001	0.022		0.0000	0.0001	0.006
	Flow-SA	-0.0001	0.0001	0.069		0.0001	0.0001	0.015

	Flow-SlogA	0.0000	0.0001	0.010		0.0000	0.0001	0.004	
NO3	Unweighted	**-0.0341**	**0.0092**	**0.282**	*******	**0.0247**	**0.0115**	**0.117**	*****
	Euclid	**-0.0202**	**0.0079**	**0.159**	*****	0.0147	0.0089	0.072	
	Euclid-S	**-0.0253**	**0.0092**	**0.178**	******	0.0201	0.0104	0.096	
	Euclid-A	-0.0047	0.0071	0.012		0.0018	0.0077	0.002	
	Euclid-logA	**-0.0229**	**0.0083**	**0.178**	******	0.0170	0.0094	0.085	
	Euclid-SA	-0.0042	0.0072	0.010		0.0014	0.0079	0.001	
	Euclid-SlogA	**-0.0229**	**0.0091**	**0.153**	*****	0.0179	0.0101	0.081	
	Flow	**-0.0257**	**0.0107**	**0.140**	*****	0.0161	0.0124	0.046	
	Flow-S	-0.0215	0.0120	0.084		0.0137	0.0135	0.029	
	Flow-A	**-0.0230**	**0.0089**	**0.161**	*****	0.0176	0.0107	0.072	
	Flow-logA	**-0.0249**	**0.0105**	**0.139**	*****	0.0160	0.0122	0.047	
	Flow-SA	**-0.0273**	**0.0099**	**0.179**	******	0.0240	0.0123	0.098	
	Flow-SlogA	-0.0210	0.0116	0.086		0.0139	0.0132	0.031	
TP	Unweighted	**-0.0007**	**0.0003**	**0.172**	*****	**0.0007**	**0.0003**	**0.127**	*****
	Euclid	**-0.0006**	**0.0002**	**0.185**	******	**0.0006**	**0.0002**	**0.140**	*****
	Euclid-S	**-0.0009**	**0.0002**	**0.311**	*******	**0.0009**	**0.0003**	**0.269**	*******
	Euclid-A	**-0.0006**	**0.0002**	**0.244**	******	**0.0005**	**0.0002**	**0.170**	*****
	Euclid-logA	**-0.0008**	**0.0002**	**0.301**	*******	**0.0008**	**0.0002**	**0.232**	******
	Euclid-SA	**-0.0005**	**0.0002**	**0.223**	******	**0.0005**	**0.0002**	**0.158**	*****
	Euclid-SlogA	**-0.0009**	**0.0002**	**0.305**	*******	**0.0009**	**0.0002**	**0.260**	******
	Flow	-0.0005	0.0003	0.084		0.0005	0.0003	0.061	
	Flow-S	-0.0005	0.0003	0.075		0.0005	0.0004	0.062	
	Flow-A	**-0.0008**	**0.0002**	**0.245**	******	**0.0006**	**0.0003**	**0.128**	*****
	Flow-logA	-0.0005	0.0003	0.085		0.0005	0.0003	0.064	
	Flow-SA	**-0.0008**	**0.0003**	**0.201**	******	**0.0007**	**0.0003**	**0.118**	*****
	Flow-SlogA	-0.0005	0.0003	0.076		0.0005	0.0003	0.066	
PO43	Unweighted	**-0.0004**	**0.0002**	**0.121**	*****	0.0004	0.0002	0.081	
	Euclid	**-0.0003**	**0.0001**	**0.144**	*****	0.0003	0.0002	0.101	
	Euclid-S	**-0.0005**	**0.0002**	**0.243**	******	**0.0005**	**0.0002**	**0.202**	******
	Euclid-A	**-0.0003**	**0.0001**	**0.184**	******	**0.0003**	**0.0001**	**0.117**	*****
	Euclid-logA	**-0.0005**	**0.0001**	**0.235**	******	**0.0004**	**0.0002**	**0.171**	*****
	Euclid-SA	**-0.0003**	**0.0001**	**0.167**	*****	**0.0003**	**0.0001**	**0.107**	*****
	Euclid-SlogA	**-0.0005**	**0.0001**	**0.239**	******	**0.0005**	**0.0002**	**0.196**	******
	Flow	-0.0003	0.0002	0.054		0.0002	0.0002	0.036	

	Flow-S	-0.0003	0.0002	0.049		0.0003	0.0002	0.040	
	Flow-A	**-0.0004**	**0.0002**	**0.186**	**	0.0003	0.0002	0.081	
	Flow-logA	-0.0003	0.0002	0.055		0.0003	0.0002	0.039	
	Flow-SA	**-0.0004**	**0.0002**	**0.150**	*	0.0004	0.0002	0.074	
	Flow-SlogA	-0.0003	0.0002	0.051		0.0003	0.0002	0.044	
A254	Unweighted	0.0008	0.0010	0.018		-0.0004	0.0011	0.003	
	Euclid	0.0011	0.0008	0.061		-0.0010	0.0009	0.037	
	Euclid-S	0.0007	0.0009	0.018		-0.0005	0.0010	0.007	
	Euclid-A	-0.0001	0.0007	0.001		0.0003	0.0007	0.004	
	Euclid-logA	0.0007	0.0009	0.019		-0.0004	0.0009	0.007	
	Euclid-SA	0.0000	0.0007	0.000		0.0001	0.0007	0.001	
	Euclid-SlogA	0.0006	0.0009	0.013		-0.0004	0.0010	0.004	
	Flow	0.0001	0.0011	0.000		0.0004	0.0012	0.004	
	Flow-S	-0.0001	0.0012	0.000		0.0006	0.0013	0.006	
	Flow-A	0.0001	0.0009	0.001		0.0005	0.0011	0.006	
	Flow-logA	0.0001	0.0011	0.000		0.0004	0.0012	0.003	
	Flow-SA	0.0002	0.0010	0.001		0.0003	0.0012	0.002	
	Flow-SlogA	-0.0001	0.0012	0.000		0.0005	0.0013	0.005	
Ca	Unweighted	**-0.2962**	**0.0609**	**0.403**	***	**0.2873**	**0.0744**	**0.299**	***
	Euclid	**-0.1872**	**0.0536**	**0.259**	**	**0.1832**	**0.0600**	**0.210**	**
	Euclid-S	**-0.2577**	**0.0594**	**0.349**	***	**0.2564**	**0.0667**	**0.297**	***
	Euclid-A	-0.0975	0.0492	0.101		0.0883	0.0543	0.070	
	Euclid-logA	**-0.2325**	**0.0538**	**0.348**	***	**0.2247**	**0.0605**	**0.283**	***
	Euclid-SA	-0.0977	0.0497	0.100		0.0897	0.0551	0.070	
	Euclid-SlogA	**-0.2425**	**0.0592**	**0.324**	***	**0.2368**	**0.0657**	**0.271**	***
	Flow	**-0.2409**	**0.0736**	**0.234**	**	**0.2087**	**0.0852**	**0.146**	*
	Flow-S	**-0.2218**	**0.0830**	**0.170**	*	**0.1948**	**0.0940**	**0.109**	*
	Flow-A	**-0.2062**	**0.0610**	**0.246**	**	**0.1842**	**0.0742**	**0.150**	*
	Flow-logA	**-0.2332**	**0.0722**	**0.229**	**	**0.2065**	**0.0837**	**0.148**	*
	Flow-SA	**-0.2364**	**0.0684**	**0.254**	**	**0.2255**	**0.0859**	**0.164**	*
	Flow-SlogA	**-0.2161**	**0.0804**	**0.171**	*	**0.1943**	**0.0915**	**0.114**	*

[a] Significance of the regression slope. Significance codes: *: $p < 0.05$, **: $p < 0.01$, ***: $p < 0.001$

Supplementary Table S5. Pearson's correlation coefficients and corresponding p-values for forests and meadows proportions measured by different metrics.

Metric	*r*	*p-value*

Unweighted	-0.960	< 0.001
Flow	-0.958	< 0.001
Flow-S	-0.970	< 0.001
Flow-A	-0.966	< 0.001
Flow-SA	-0.971	< 0.001
Flow-logA	-0.957	< 0.001
Flow-SlogA	-0.969	< 0.001
Euclid	-0.980	< 0.001
Euclid-S	-0.986	< 0.001
Euclid-A	-0.976	< 0.001
Euclid-SA	-0.974	< 0.001
Euclid-logA	-0.980	< 0.001
Euclid-SlogA	-0.988	< 0.001
